# Novel Liver Parenchymal Transection Technique Using Saline-linked Monopolar Cautery Scissors (SLiC-Scissors) in Robotic Liver Resection

**DOI:** 10.7759/cureus.28118

**Published:** 2022-08-17

**Authors:** Takahisa Fujikawa, Yusuke Uemoto, Taisuke Matsuoka, Masatoshi Kajiwara

**Affiliations:** 1 Surgery, Kokura Memorial Hospital, Kitakyushu, JPN; 2 Surgery, Kokura Memorial Hospital, Kitakyushu, Fukuoka, JPN; 3 Gastroenterological Surgery, Faculty of Medicine, Fukuoka University, Fukuoka, JPN

**Keywords:** liver surgery, hepatocellular carcinoma, liver metastasis, liver parenchymal transection, robotic liver resection

## Abstract

Introduction

Although there are a number of benefits to using robotics in liver surgery over conventional open and laparoscopic approaches, liver parenchymal transection is still the most difficult aspect of robotic liver resection (RLR) due to the limitations of the currently available robotic instruments and the lack of a standardized method.

Methods

We present a novel method for transecting the liver parenchyma during RLR employing saline-linked monopolar cautery (SLiC) scissors (SLiC-Scissors method). Between September 2021 and April 2022, 10 RLRs were performed utilizing the SLiC-Scissors method for both anatomical and non-anatomical liver resections. We assessed the short-term results, as well as the safety and practicality of our robotic liver parenchymal transection technique.

Results

Six of the 10 patients had malignant liver tumors, and four of them had liver metastases from colorectal cancer. Except for S1, the target lesions were present everywhere, and their median size was 25 mm (14-43 mm). The median amount of intraoperative bleeding was 5 mL (5-30 mL), and the median operative and console times were 223 and 134 min, respectively. There were no conversions to open liver resections. The median length of the postoperative stay was seven (4-13) days, and there were no serious postoperative complications or mortality.

Conclusions

The SLiC-Scissors method is a safe and practical procedure for liver parenchymal transection in RLR. In order to standardize and broadly implement RLR into normal patient treatment, this unique approach enables an advanced, locally controlled preparation of intrahepatic vessels and bile ducts.

## Introduction

Minimally invasive surgery has been implemented in liver surgery with continued success [1-6] and laparoscopic liver resection (LLR) has been performed more frequently, although laparoscopic major liver resection still appears to be challenging due to the intrinsic constraints of laparoscopic procedures, such as the fulcrum effect and the limited degrees of devices. Recently, robotic liver resections (RLRs) have gained acceptance and expanded indications [5-8]. These can be safely carried out with less blood loss, quicker recovery from surgery, and less postoperative pain compared to open operations while preserving comparable oncological results [4, 7-13].

Intuitive hand-control motions, tools with seven degrees of freedom, a magnified three-dimensional perspective, and steady robotic arm retraction are all features of the robotic systems that have been developed to enhance a surgeon's skills [14]. On the other hand, liver parenchymal transection is one of the most difficult stages of RLR since there is not enough equipment available for it. There are currently no standards, and various procedures for parenchymal transection have been documented [4, 13, 15].

In this paper, we provide a unique method for robotic liver parenchymal transection employing saline-linked monopolar cautery (SLiC) scissors, which are as efficient and secure as LLR.

## Materials and methods

A total of 10 RLRs performed at our institution between September 2021 and April 2022 were included in this study. Of those, eight partial liver resections and two anatomical resections (one left lateral sectionectomy and one S3 subsectionectomy) were included. We have introduced RLR based on the sufficient preparation of the surgical team, and all procedures were performed by one board-certified attending surgeon (TF). We started our robotic liver program by selecting patients qualified for limited resection. Currently, only patients with liver tumors 10 cm or larger in size or those with tumors being considered for vascular reconstruction or multi-visceral resection are excluded from RLR.

Demographics, diagnoses, surgical treatments, and postoperative outcomes were obtained through a standardized review of the electronic surgery database as well as hospital and clinic charts. The information was gathered prospectively and analyzed retrospectively. We evaluated the short-term outcomes as well as the safety and feasibility of our method of robotic liver parenchymal transection. Postoperative complications were categorized and assessed using the Clavien-Dindo classification (CDC) [16], and CDC class II or higher was considered significant. Operative mortality included death within 30 days after surgery. The study protocol complied with the Declaration of Helsinki and was approved by the Kokura Memorial Hospital Clinical Research Ethics Committee (#21021002).

Surgical technique

The patient was placed supine in a 10° reverse Trendelenburg position with the legs apart, and the operating table tilted slightly to the left in the case of a right lobe tumor. Alternatively, the patient was placed in the left semi-flank position for resection of right posterosuperior segment tumors. The first trocar was inserted through an incision made on the top of the umbilicus. The pneumoperitoneum was achieved with the Hasson method, and intraabdominal pressure was maintained at 8-12 mmHg.

**Figure 1:**
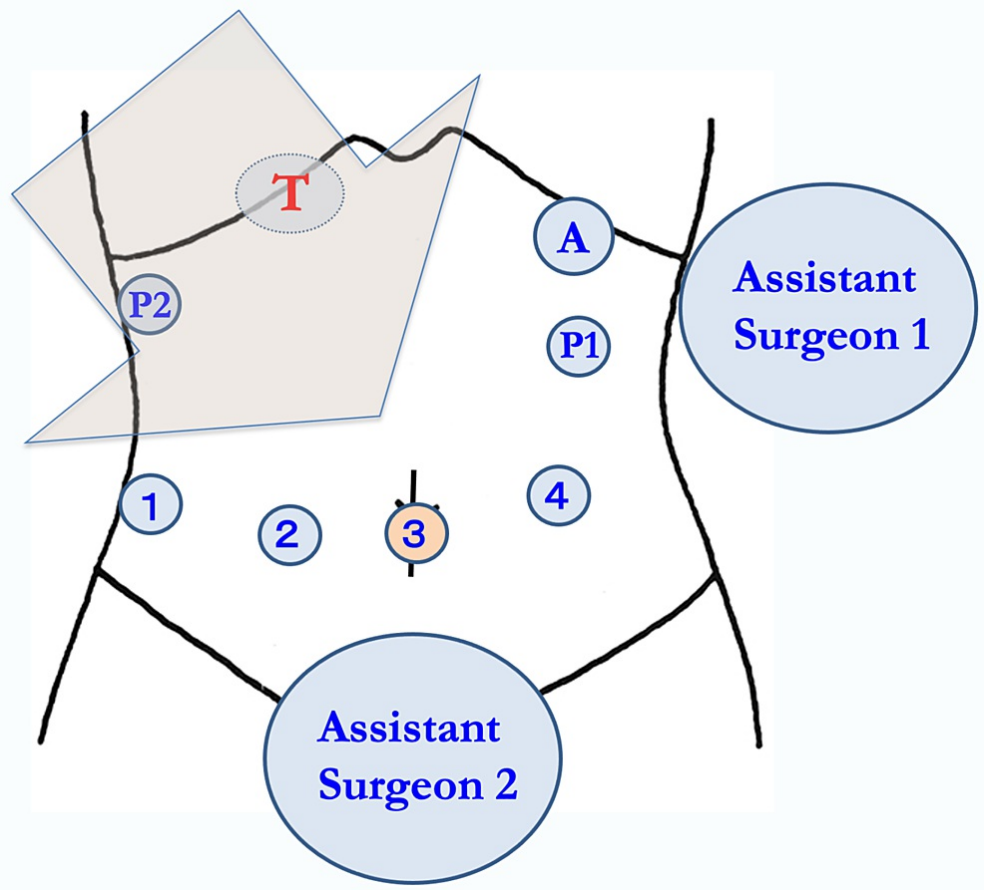
Trocar placement for robotic liver resection Five trocars are typically used for the procedure and were placed concentrically around the targeted liver lobe, which illustrates four trocars for robotics and one 12-mm trocar from the left-upper side for the assistant surgeon. The patient cart was rolled in and placed from the right cranial side of the patient. The assistant surgeon stood on the patient's left side, while the second assistant surgeon was positioned between the patient's legs. T: the targeted lobe of the liver; A: 12-mm trocar for the assistant surgeon; 1,2,3,4: the four robotic trocars; P1: Pringle maneuver for the right lobe tumor; P2: Pringle maneuver for the left lobe tumor; big colored arrow: role-in direction of the patient cart.

All 10 RLRs in our series were performed using the Da Vinci Xi surgical system (Intuitive Surgical, Inc., Sunnyvale, CA, USA). Five trocars were typically used for the procedure and were placed concentrically around the targeted liver lobe as shown in Figure 1, which illustrates four trocars for robotics and one 12-mm trocar from the left-upper side for the assistant surgeon. The patient cart was rolled in and placed from the right cranial side of the patient. The first surgeon was seated at the robotic console, while the assistant surgeon stood on the patient's left side. The second assistant surgeon was positioned between the patient's legs (Figure 1).

No. 1, 2, 3, and 4 robotic trocars were primarily assigned to liver retraction (the third arm), the surgeon’s left hand, the endoscope, and the right hand, respectively. The No. 1, 2, and 4 arms were generally operated using Cadiere or Tip-up Forceps®, Fenestrated Bipolar Forceps®, and Maryland Bipolar Forceps®, respectively, although EndoWrist Suction Irrigator® and monopolar curved scissors were placed on the left and right hands, respectively, during liver parenchymal transection. Before parenchymal transection, the Pringle maneuver was prepared through the extraperitoneal tourniquet system, which was placed in the left or right mid-upper abdomen for right or left lobe tumors, respectively, and used on demand.

SLiC-Scissors method for liver parenchymal transection

During liver parenchymal transection, the console surgeon's forceps used monopolar cautery curved scissors for the right hand (robotic arm 4) and a Suction Irrigator® for the left hand (robotic arm 2), and the third arm was towed and fixed with Tip-up or Cadiere Forceps® (robotic arm 1). The monopolar cautery scissors were plugged into the integrated ERBE VIO dV (ERBE USA, Marietta, GA) with Forced Coag mode (effect 1, power limit 40-50 W), in which the electrosurgical performance was similar to the soft coagulation mode in the VIO device. The assistant surgeon prepared to handle the ball-tipped SLiC, which was plugged into an electrosurgical VIO device (ERBE Elektromedizin GmbH, Tübingen, Germany) and was linked to a sterile 0.9% saline bottle, and the drip rate was set at 1-2 cc/minute. During the parenchymal transection, intraparenchymal vessels smaller than 2 mm were dissected by scissors, but vascular structures with a diameter greater than 2 mm were clipped and/or ligated and divided by ultrasonic coagulating shears (CS; Ethicon, Cincinnati, Ohio) by the assistant surgeon. In the case of dissecting major glissonean pedicles, the waterjet scalpel (Erbejet® 2, Erbe Elektromedizin GmbH, Tuebingen, Germany) was used as a supplement from the assistant surgeon’s side to avoid heat injury to the bile ducts.

**Figure 2:**
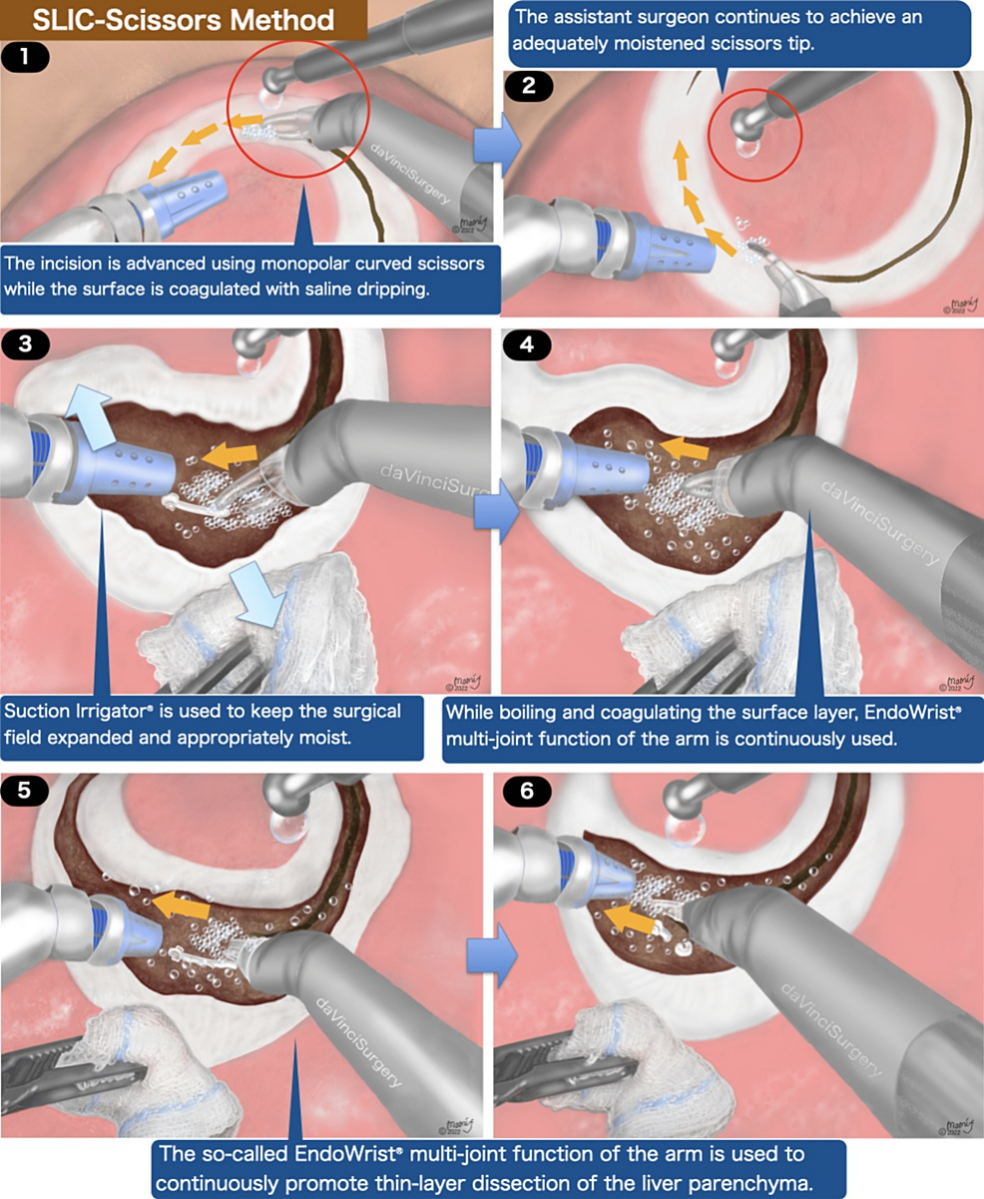
The process of robotic liver parenchymal transection via the SLiC-Scissors method (Part 1) SLiC-Scissors: saline-linked electrocautery scissors.

**Figure 3:**
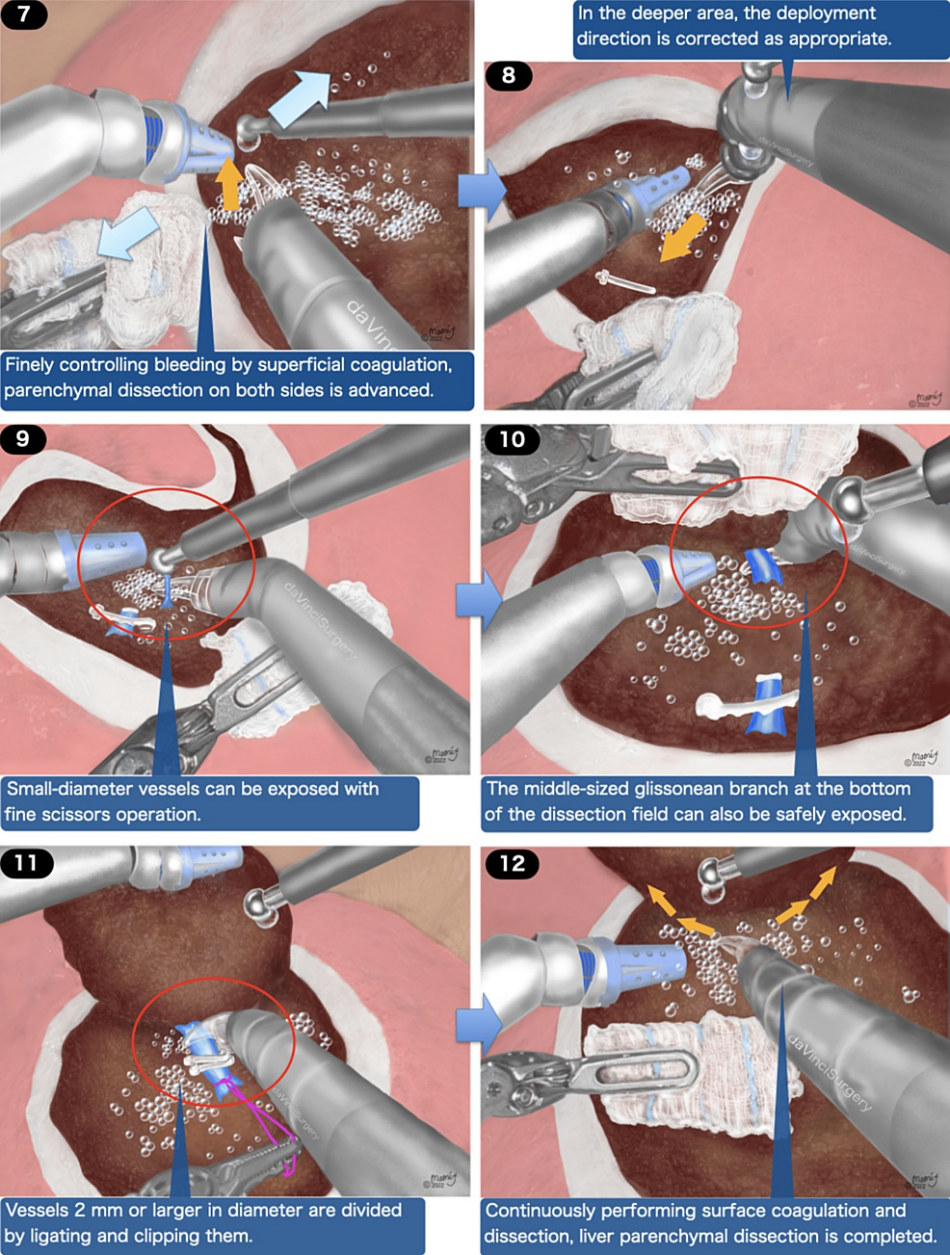
The process of robotic liver parenchymal transection via the SLiC-Scissors method (Part 2)

Figures 2, 3, and Video 1 depict the general framework of the SLiC-Scissors method. The dissection and hemostasis were advanced while low-temperature (up to 100 degrees Celsius or below) heat coagulation of the superficial layer of the dissection surface was performed by dripping saline droplets from the assistant side. In the beginning, the incision was advanced using monopolar cautery scissors while the surface was coagulated with saline dripping. The assistant surgeon continued to achieve an adequately moistened scissors tip. The Suction Irrigator® was used to keep the surgical field expanded and appropriately moist. While boiling and coagulating the surface layer, the EndoWrist® multi-joint function of the arm was continuously used. The EndoWrist® monopolar cautery scissors were used to continuously promote thin-layer dissection of the liver parenchyma.

**Video 1:** The outline of the SLiC-Scissors method in robotic liver resection (with narration) This video provides a narrated summary of the SLiC-Scissors method. SLiC-Scissors: saline-linked electrocautery scissors.

Parenchymal dissection on both sides was advanced while superficial coagulation was applied to precisely control hemorrhage. The Endowrist® function might adjust the deployment direction in the deeper area as necessary. The small-diameter vessels could be exposed with a fine scissor motion, and the middle-sized glissonean branch (2mm or larger in diameter) near the bottom of the dissection field was split by ligating and clipping. In the last phase, continuous surface coagulation and dissection were conducted, and then the liver parenchymal dissection was completed (Video 2).

**Video 2:** The SLiC-Scissors method in robotic liver resection This video provides a summary of the SLiC-Scissors method for liver parenchymal transection during robotic hepatectomy. SLiC-Scissors: saline-linked electrocautery scissors.

## Results

To assess the efficacy and safety of our approach using the SLiC-Scissors method for robotic liver parenchymal transection, we assessed 10 initial consecutive cases undergoing RLR using SLiC-Scissors. The background characteristics of 10 included cases are shown in Table 1. Included in the 10 cases were colorectal cancer liver metastasis in four cases and liver malignant tumors in six cases. Three of the patients had liver cirrhosis, and two of the patients had liver metastases from colon cancer that required hepatectomy following chemotherapy and dissection of the main lesion.

**Table 1:** Background characteristics of patients undergoing robotic liver resection using SLiC-Scissors method SLiC-Scissors: saline-linked electrocautery scissors; BMI: body mass index; Preop.: preoperative; CRC-LM: colorectal cancer liver metastasis; HCC: hepatocellular carcinoma.

No.	age, year	Gender	BMI, kg/m2	Disease	Maximal tumor size, mm	Multiple	Liver cirrhosis	Preop. chemotherapy
1	78	male	21.3	CRC-LM	14	No	No	Yes
2	73	male	20.1	CRC-LM	25	No	No	Yes
3	75	male	20.1	HCC	27	No	Yes	No
4	68	male	21.3	Liver tumor	25	No	No	No
5	73	male	25.2	HCC	18	No	No	No
6	72	male	21.2	CRC-LM	24	Yes	No	No
7	64	male	22.4	CRC-LM	20	Yes	No	No
8	77	male	24.5	HCC	35	No	Yes	No
9	73	female	26.5	HCC	43	No	Yes	No
10	75	male	22.9	Liver tumor	25	No	No	No

Table 2 displays the tumor characteristics and surgical outcomes of the enrolled patients. The target lesions were situated everywhere except S1 (S2 (n=3), S3 (n=2), S4/5 (n=1), S5 (n=2), S6 (n=2), S7 (n=1), and S8 (n=1)), and their median size was 25 mm (14-43 mm). The median amount of bleeding was 5 mL (5-30 mL), and the median operating and console times were 223 (143-433) and 134 (72-261) min, respectively. There were no cases of conversion to open liver resection. There were no severe complications throughout the postoperative period, and there was no mortality. The median length of postoperative stay was seven (4-13) days.

**Table 2:** Tumor characteristics and surgical outcome of patients undergoing robotic liver resection using SLiC-Scissors method SLiC-Scissors: saline-linked electrocautery scissors; LOS: length of postoperative stay.

No.	Tumor location, size	Procedure	Operative time, min	Docking time, min	Blood loss, mL	Morbidity	LOS, days
1	S6, 14mm	Limited resection, adhesiolysis	260	112	5	Cholangitis	10
2	S2, 25mm	Limited resection	211	128	5	None	6
3	S6, 27mm	Limited resection	234	139	5	None	5
4	S4/5, 25mm	Limited resection, cholecystectomy	143	72	5	None	4
5	S8, 18mm	Limited resection	212	104	5	Delirium	10
6	S3, 24mm / S5, 18mm	Limited resection	189	83	15	None	5
7	S2, 20mm / S5, 17mm	Limited resection, adhesiolysis, cholecystectomy	433	258	30	None	9
8	S2/3, 35mm	Lateral Sectionectomy	364	261	5	None	5
9	S3, 43mm	S3 Subsectionectomy	259	166	9	None	13
10	S7/6, 25mm	Limited resection	211	139	5	None	8

## Discussion

The novel approach of the SLiC-Scissors method during RLR is described in the current article. Using this strategy, rapid dissection and hemostasis can be achieved simultaneously during RLR. Additionally, it might be done without increasing the risks of bile leakage or bile duct stenosis due to heat damage. Consequently, the SLiC-Scissors method can deliver safe and time-efficient liver parenchymal transection during RLR.

Minimally invasive liver resections have become more popular in recent years due to their perioperative benefits over open liver surgery. Although LLR is widely used in minor and major liver surgery, RLR offers several benefits over traditional laparoscopy [8, 14]. The so-called EndoWrist®, which enables the handling of the instrument tips in seven degrees of freedom, stable three-dimensional visualization, tremor filtration with motion scaling capacity, and the maneuvering of three instruments by the console surgeon, are all just some of the technical advantages highlighted in a recently published international consensus statement [8]. These elements allow for an accurate vessel dissection during RLR. With the oncologic result being equivalent to open and laparoscopic operations, RLR is currently recognized as a safe and practicable treatment [8].

Although robotics allows for thorough dissection of major glissonean pedicles or hepatic veins, current data indicate that transection of the liver parenchyma is the main restriction of RLR. According to a recent study, there are fewer equipment options for RLR than for laparoscopic or open procedures [3]. Giulianotti et al. first suggested the technique in which monopolar scissors and bipolar forceps were utilized with suture retraction [13] for the approach for liver parenchymal transection during RLR. However, because the necrotic, coagulated tissue is frequently stuck to the tip of the scissors or forceps, preventing precise hemostasis, this technique could not completely control bleeding during parenchymal transection. The methods suggested by Choi et al. [4], which employ Maryland forceps and ultrasonic coagulating shears on both robotic hands, also have a number of drawbacks. The lack of EndoWrist® function, which elevates the risk of significant damage to major intrahepatic vessels in the deep parenchymal layers, is the principal limitation of coagulating shears transection. The new Endowrist Vessel Sealer® was just released, however, because of the thickness of its tip, it appears to have some of the same limitations for efficiently transecting the liver parenchyma [4]. As a result, alternative strategies must be developed to promote satisfactory RLR.

Following our experience and altering the methodology used in laparoscopic surgeries, we described a novel approach to robotic liver parenchyma dissection utilizing the SLiC-Scissors method. This method combines the benefits of the existing instruments for robotic and laparoscopic surgery. Originally, we developed a "modified two-surgeon technique" for liver parenchymal transection during LLR [17] based on the "Kyoto University-style liver parenchymal transection" procedure during open liver resection [18, 19], in which rapid laparoscopic liver parenchymal transection comparable to open liver resection can be achieved by thoroughly sharing the roles. The multifaceted "ball-rolling" motion of the saline-linked ball-tipped monopolar cautery can control the majority of bleeding sites in the "hemostatic mode" of this procedure. We also use the saline-linked cautery combined with wet oxidized cellulose (SLiC-WOC) [20], which allows the majority of uncontrollable bleeding from the deep liver parenchymal fissure to be quickly managed laparoscopically. It is possible to successfully transport superficial boiling heat coagulation by contact with sodium chloride solution to the bleeding point by properly moistening the surgical field's surface or inserting wet oxidized cellulose.

Through the use of monopolar cautery curved scissors in conjunction with continuous saline dripping, our cutting-edge SLiC-Scissors approach also provides superficial heat coagulation. By applying low-temperature heat coagulation (up to 100 degrees Celsius or below) to the superficial layer by dropping saline droplets from the assistance side, the fast dissection and hemostasis can be expedited. The instruments remain clean using saline, and necrotic or coagulated tissue rarely needs to be removed from the tip of the robotic instruments. Additionally, the robotic systems include tremor filtration and motion scaling capabilities, which enable meticulous manipulation of the sharp-edged scissors and the implementation of safe thin-layered liver parenchymal transection and hemostasis.

There are certain restrictions on the current study. First, because the study was conducted retrospectively, it is only of limited use in determining how treatments affect outcomes. Second, the sample size was insufficient, and a bigger sample size would likely be beneficial in generating more trustworthy recommendations. Finally, in order to demonstrate that our recommended strategy is superior to alternatives, a control group is necessary. We are convinced that this method is a step toward future RLR standardization. It may lead to improved security and outcomes and constitute a further step toward the widespread implementation of RLR.

## Conclusions

SLiC-scissors method for liver parenchymal transection in RLR is a safe and feasible procedure. With this novel approach, the intrahepatic vessels are better exposed and local control is improved. This approach is becoming a crucial step toward the standardization of RLR.
